# Is this Judaism? The Question of the Consistency of Israeli Policy and Actions in Gaza with Jewish Thought and Ethics: Part 2: Evidence, Analysis and Conclusions

**DOI:** 10.1007/s11673-025-10488-7

**Published:** 2025-09-11

**Authors:** P. A. Komesaroff, J. Z. Kenner

**Affiliations:** 1 Monash University, Melbourne, VIC Australia; 2 The National Health and Medical Research Council of Australia, Canberra, Australia

**Keywords:** Ethics, War, Conflict, Judaism, Talmud, War crimes, Human shields, Self-defence, Bible, Religion, Collective punishment, Israel, Gaza, Palestine, IDF, Hamas, Geopolitical ethics

## Abstract

There has been much discussion about the tactics used by the Israeli Defence Forces (IDF) and government in the conflict in Gaza following October 7, 2023, which have caused, among other things, systematic destruction of hospitals and schools, the deaths of large numbers of civilians, including women and children, mass starvation, and denial of humanitarian aid. The Israeli government and IDF have sought to justify their actions using ethical arguments, many of which relate to their proclaimed role as the representatives of the Jewish state and of Jewish culture and history. Arguing from the extensive corpus of Jewish ethical thought, extending back thousands of years, this article poses a simple question: Are the above actions by the Israeli government and IDF in Gaza consistent with the ethical tradition of Judaism and the obligations that flow from it? To answer this question, key texts are analysed, especially the Hebrew Bible and the Talmud, and multiple arguments are examined, taking into account the complexities of context and diverse interpretive theories. The paper is presented in two parts, the first discussing the question and methodological issues and the second providing the data and conclusions. We conclude that the alleged acts of the Israeli government and IDF in Gaza are clearly and directly contrary to the Judaic tradition of ethics as it has developed over the millennia. The conduct of the war cannot truthfully be presented in any meaningful sense as representing, or indeed, consistent with, Jewish culture or ethics. These findings have potentially far-reaching consequences, including for the claimed status of Israel as a Jewish state, the relationship between criticism of the government of Israel and the scourge of antisemitism, and the identity of Jewish people both within and outside Israel.

In part 1 of this essay we laid out the question we are seeking to answer and examined in detail the methodological and hermeneutic issues associated with it. Specifically, we stated the question in the following terms: Are the acts of the Israeli government and the IDF consistent with the philosophy and ethics of Judaism? We identified as authoritative texts those contained in the Hebrew Bible and the rabbinical literature, including the Talmud and subsequent commentaries.

In part 2 we shall examine the evidence supporting both positive and negative answers to this question.

## Possible Ways of Justifying the Actions of the IDF

The violence and ferocity of the Israeli response is not contested. What has been questioned is whether this response can be justified ethically in a manner consistent with the Judaic tradition. Justifications have been proposed by the Israeli government and its supporters in Israel and overseas.

These justifications have taken two main forms. On the one hand, members of the government, including the Israeli Prime Minister himself as well as several of his ministers, have repeatedly referred to passages of the Bible as a basis for the policies and actions they have followed. These have been supported by statements by other parties, including Rabbis, cultural leaders, and health professionals. The biblical passages are presented as if they provide authoritative evidence in support of the actions undertaken by the IDF. The existence of such textual references do not formally constitute “arguments” in a philosophical (or even a doctrinal) sense; however, they do implicitly make claims about a close and harmonious relationship between the Judaic corpus and the actions in question.

On the other hand, Israeli government officials and religious and cultural leaders have invoked more contemporary forms of ethical reasoning: for example, relying on claims that Israel’s response to the Hamas attack was broadly justified by a right to self-defence, that attacks on civilians were also justified because the civilians were being used as human shields, and that Israel was, overall, acting responsibly and with appropriate proportionality.

Examples of the former style of justification include Benjamin Netanyahu’s famous speech on October 28, 2023 which cited a passage from Deuteronomy 25:17: “You must remember what Amalek has done to you, says our Holy Bible” (Ministry of Foreign Affairs 2023). This reference to Amalek, a biblical enemy of the Israelites, has been generally understood as a justification for aggressive actions against perceived adversaries.

This oft-quoted passage is undoubtedly a reflection of the growing influence of the so-called ultra-orthodox religious community in the Israeli government, which has led to increased integration of religious narratives into military contexts (Ali 2024). Many similar claims have been made that passages in the Bible support, or are consistent with, the actions of the IDF in Gaza.

Another typical example of such thinking is the case of Rabbi Eliyahu Mali, an Israeli Rabbi who urged the deliberate killing of women and children in the Gaza Strip and said he considered it a response to the teachings of halakha, or Jewish law:

In our mitzvah [holy] war, in our situation in Gaza, according to what the law says, “Not every soul shall live,” and the logic of this is very clear: if you do not kill them, they will kill you …This means that this rule (do not keep alive every soul) is very clear in its concept, either you or them…Whoever comes to kill you, kill him first … [which does] not only include the young man aged 16, 18, 20, or 30 who is now pointing a weapon at you, but also the future generation (the children of Gaza), and those who produce the future generation (women of Gaza), because there is really no difference. (Anadolu Agency 2024)

Examples of the application of more contemporary forms of ethical reasoning include the call by a group of Israeli doctors for the bombing of hospitals in Gaza. In a letter addressed to the Israeli government and military, these physicians argued that hospitals in Gaza have been utilized by armed groups, thereby justifying military action against them. The letter states that “the residents of Gaza” have “brought their annihilation upon themselves” for allowing hospitals to become “terrorist nests” (Shapiro and Ofir 2023).

In a similar vein, StandWithUs, which presents itself as “an international, non-partisan education organization that supports Israel and fights antisemitism,” argues that:

While Israel does all it can to protect Israelis during war, Hamas and other terrorist groups use Palestinians as human shields. As a result, Palestinians tend to suffer far more civilian casualties than Israelis do. Some in the international community exploit this to make deeply misleading accusations about IDF “war crimes” and “disproportionate force” ... Proportionate force “cannot in any way be determined by considering the relative casualty figures between belligerents in a conflict.” Proportionality is determined by weighing the benefit of a strike against the potential harm to civilians. This can be extremely difficult to decide in the heat of battle ... Attacks targeting legitimate military objectives that unintentionally result in civilian deaths or injuries are not war crimes. (StandWithUs 2024)

Other examples of both strategies for justification of Israeli government and IDF actions abound (Hayford 2023; Kasher 2009; Kuttab 2023).

## Potential Biblical Justifications for Israeli Actions

A careful reading of the Bible does indeed provide apparent textual support for at least some of the acts and policies referred to. Multiple passages relate to conduct in war, and the responses of the Jewish people to external threats. They include claims about unrestricted ownership of the land of Israel, acceptance or promotion of a policy of driving out and killing all opponents, justification for acts of retribution, and defence against offences of various kinds.

The following are examples of such claims. Justifications, and exhortations:

(i) The promised land and the divine covenant

Multiple references can be said to support a purported divine mandate for the Jewish people to inhabit and control the land of Israel, including both the contemporary regions commonly referred to as Gaza and the West Bank and broader areas of land referred to as “Greater Israel” by proponents of this claim. These argue that the land was divinely promised to the descendants of Abraham^1^ and that contemporary actions to secure it are a continuation of the project of fulfilling that covenant:

- **Genesis 12:7:** “To your offspring I will give this land.”
- **Genesis 15:18-21**: “To your descendants I give this land, from the Wadi of Egypt to the great river, the Euphrates.”
- **Deuteronomy 1:8**: “See, I have given you this land. Go in and take possession of the land that the Lord swore he would give to your fathers.”

(ii) Commandments to drive out inhabitants

A number of verses are interpreted to justify aggressive or militaristic actions against perceived adversaries, drawing parallels between ancient conflicts in the Old Testament and modern struggles for territorial control. Critics argue that such interpretations are taken out of historical and cultural contexts:

- **Numbers 33:50–53**: “Drive out all the inhabitants of the land before you. Destroy all their carved images and cast idols and demolish all their high places. Take possession of the land and settle in it.”
- **Deuteronomy 7:1–2**: “When the Lord your God brings you into the land you are entering to possess ... you must destroy them totally. Make no treaty with them and show them no mercy.”
- **Deuteronomy 20:16–18**: “In the cities of the nations the Lord your God is giving you as an inheritance, do not leave alive anything that breathes. Completely destroy them ... as the Lord your God has commanded you.”
- **Joshua 6:21**: “They devoted the city to the Lord and destroyed with the sword every living thing in it—men and women, young and old, cattle, sheep, and donkeys.”

(iii) Restoration of Israel

Some verses are often linked to the establishment of the modern State of Israel in 1948 and the idea that its continued existence and expansion are the fulfilment of biblical prophecy. This perspective sees territorial actions as part of the divine restoration of Israel:

- **Ezekiel 37:21–22**: “This is what the Sovereign Lord says: I will take the Israelites out of the nations where they have gone. I will gather them from all around and bring them back into their own land.”
- **Isaiah 66:8**: “Can a country be born in a day or a nation be brought forth in a moment? Yet no sooner is Zion in labour than she gives birth to her children.”

(iv) The right of self-defence

Some biblical texts are used to argue that self-defence, even using violent means, is ethically justified when the security of the nation or its people is threatened. In this manner, the attacks by Hamas are framed as an existential threat to Israel, thereby legitimizing strong retaliatory measures under the principle of divine sanction for protection:

- **Exodus 22:2**: “If a thief is caught breaking in at night and is struck a fatal blow, the defender is not guilty of bloodshed.”
- **Leviticus 24:19–20**: “If anyone injures his neighbour, as he has done it shall be done to him, fracture for fracture, eye for eye, tooth for tooth.”

(v)  The pursuit of justice

Some verses are interpreted to mean that “justice” must be served against those who commit acts of violence or terror. Hamas’s actions, including the killing of civilians, are viewed as violations of justice that necessitate a proportional or decisive response to restore the moral and social order:

- **Deuteronomy 16:20**: “Justice, and only justice, you shall follow, that you may live and inherit the land that the Lord your God is giving you.”
- **Proverbs 21:15**: “When justice is done, it brings joy to the righteous but terror to evildoers.”

(vi) An obligation to eradicate evil

Some texts are cited to justify the eradication of perceived evil, in this case, the threat posed by Hamas. The destruction of Hamas infrastructure and personnel is framed as a divinely sanctioned act to remove evil influences and ensure the safety of Israel:

- **Deuteronomy 20:16–18:** “But in the cities of these peoples that the Lord your God is giving you for an inheritance, you shall save alive nothing that breathes, but you shall devote them to complete destruction ... as the Lord your God has commanded, that they may not teach you to do according to all their abominable practices.”
- **Isaiah 34:2–5:** “The Lord is enraged against all the nations ... Their slain shall be cast out, and the stench of their corpses shall rise.”

(vii) Protection of the Promised Land

Some texts are used to support the claim that Israel has a divine right and obligation to protect its territory. Any force used to safeguard the land and its people is seen as fulfilling God’s covenant with Israel:

- **Deuteronomy 1:8:** “See, I have set the land before you. Go in and take possession of the land that the Lord swore to your fathers, to Abraham, to Isaac, and to Jacob, to give to them and to their offspring after them.”
- **Numbers 33:53–54:** “You shall take possession of the land and settle in it, for I have given the land to you to possess it ... You shall inherit the land by lot according to your clans.”
- **Deuteronomy 7:1–2:** “When the Lord your God brings you into the land that you are entering to take possession of it, and clears away many nations before you ... you must devote them to complete destruction. You shall make no covenant with them and show no mercy to them.”

(viii) Retribution

In some passages retribution appeared to be framed as a moral obligation, drawing on principles of proportional justice. Supporters argue that Israel’s response to the attacks is in line with biblical precedents of ensuring consequences for aggressors:

- **Exodus 21:24**: “Eye for eye, tooth for tooth, hand for hand, foot for foot, burn for burn, wound for wound, stripe for stripe.”
- **1 Samuel 15:3**: “Now go, attack the Amalekites and totally destroy all that belongs to them.”

(ix) Divine protection for the People of Israel

Multiple verses emphasize the unique relationship between God and Israel. Supporters argue that violent actions are justified in protecting a nation and people under divine covenant, with the assurance that such measures align with God’s will:

- **Numbers 10:9:** “When you go to war in your land against the adversary who oppresses you, you shall sound an alarm ... and you shall be remembered before the Lord your God, and you shall be saved from your enemies.”
- **Chronicles 20:15:** “Thus says the Lord to you: ‘Do not be afraid and do not be dismayed at this great horde, for the battle is not yours but God’s.’”

This selection of quotations is not intended to imply that no countervailing or opposing claims could be made on the basis of biblical texts. On the contrary, there are many passages that support more nuanced and less truculent approaches to conflict. For example, there are commands to care for the stranger, widow, and orphan (e.g., Exodus 22:21, Leviticus 19:34) and indeed the famous passage in Leviticus 19:18:

- “You shall not take vengeance or bear a grudge against the sons of your own people, but you shall love your neighbour as yourself: I am the Lord.”

In addition, it is accepted among biblical scholars that the texts reflect specific historical, cultural, and theological contexts that may not directly apply to modern geopolitical situations, thereby also mitigating their potential contemporary application. Accordingly, a reliance on such passages to argue for a particular political outcome is inevitably subject to the allegation of selective quotation for tendentious purposes. Nonetheless, as the quotations presented show, it is not difficult to find in the Bible statements that may be taken to support an aggressive and uncompromising approach to a violent conflict.

## Talmudic Perspectives and Value^2^

Do these quotations, interpreted in the manner above, validly present the ethical views of Judaism? As mentioned, not only are multiple interpretations of the biblical texts possible but it is also the case that the accumulated body of wisdom in Judaism is far more extensive than the Bible alone. Indeed, the Talmudic texts and other rabbinical commentaries largely consist of highly nuanced interpretations of biblical and other texts, often developed in relation to the specific challenges presented by daily experiences or historical circumstances. This allowed the body of thought to evolve as an expanding, living archive of knowledge by providing for a continuing learning process in response to experiences accumulated over a long period of time.

As previously suggested, for all its widely varying claims and proclamations, the Talmud is committed to a number of abiding, foundational values, which pervade many, if not all, of its claims. Among the most prominent of these are a commitment to the sanctity of life, the prioritization of peace, mutual responsibility and compassion, and the irreducible role of respectful, rational dialogue as the organon of concepts and practices regarding values.

(i) The sanctity of human life

The Talmudic passage in Yoma 85b discusses the principle of *pikuach nefesh* (the sanctity and preservation of human life) and its precedence over almost all other religious obligations. The passage specifically addresses the question of whether saving a life overrides the observance of the Sabbath. It concludes that preserving human life takes priority, even if it requires violating Sabbath laws:

- **Yoma 85b**: “From where do we know that saving a life overrides the Sabbath? As it is written: ‘You shall keep My statutes and My ordinances, which a person shall do and live by them’ (Leviticus 18:5). The Torah says ‘live by them’ and not die by them.”

This teaching underscores the supreme value of human life in Jewish law. The phrase “live by them” is interpreted to mean that the commandments are meant to enhance life, not endanger it. Therefore, if observing a mitzvah (commandment) would jeopardize someone’s life, it is set aside to preserve life.

The principle of *pikuach nefesh* prioritizes saving life above almost all other commandments. The Talmud places the highest value on preserving life, even allowing the suspension of most commandments to save a single life. In accordance with this principle, it may be inferred that because hospitals, schools, and food distribution centres are institutions directly tied to the preservation of life they must be protected, even when following other commandments.

(ii) Pursuing peace and reconciliation

The Talmudic and rabbinic texts contain numerous teachings that emphasize the importance of peace and reconciliation. The pursuit of peace is a core Talmudic value. It is taught that even in situations of conflict, one must seek ways to resolve violence with minimal harm to all parties involved. Core values include pursuing peace, loving others, and supporting reconciliation. As understood in these texts, peace is not just a passive ideal but something to be actively sought and cultivated. Loving others, building relationships, and fostering goodwill are essential to creating harmony. The obligation to avoid unnecessary conflict is fundamental, and even in situations of potential conflict efforts must be made to resolve disputes peacefully:

- **Pirké Avot (Ethics of the Fathers) 1:12:** “Be of the disciples of Aaron, loving peace and pursuing peace, loving people and drawing them near to the Torah.”

The following passage from Maimonides’ codification of Jewish law, the *Mishneh Torah,* discusses the laws of war and peace, particularly in the context of interactions with non-Jewish nations:

- **Hilchot Melachim (Laws of Kings) 6:7:** “One does not wage war against anyone in the world until they are first offered peace. This applies both to optional wars and obligatory wars, as it is said: ‘When you approach a city to wage war against it, you shall propose peace to it’ (Deuteronomy 20:10). If they accept peace and agree to the seven Noahide laws, they are not to be harmed.”

This passage highlights the obligation to seek peace before resorting to conflict. Even in the context of war, the Torah itself mandates offering terms of peace to the opposing side. This reflects a broader value of prioritizing reconciliation and peaceful coexistence whenever possible.

(iii) The principle of responsibility and compassion for others

The principle of mutual responsibility (Rudman 2009) is explicitly stated in the Talmud:

- **Tractate Shevuot 39a**: “All of Israel are responsible for one another.”

This teaching underscores the idea that the Jewish people are interconnected and share a collective responsibility for one another’s well-being, both spiritually and materially. It implies that individuals must care for and support each other, ensuring that no one is left behind or neglected.

While the above quotation refers specifically to obligations to Jewish people there are multiple examples where the principle is extended to all others. For example:

- **Sanhedrin 37a:** “Whoever saves a single life is considered to have saved an entire world.”
- **Gittin 61a**: “One sustains poor gentiles along with poor Jews, and one visits sick gentiles along with sick Jews, and one buries dead gentiles along with dead Jews. All this is done on account of the ways of peace.”

The Talmud places great emphasis on acts of kindness and compassion (Nunnally 2012). For example, referring to the fact that the Torah starts with God clothing Adam and Eve (Genesis 3:21) and ends with God burying Moses (Deuteronomy 34:6):

- **Tractate Sotah 14a:** “The Torah begins with acts of kindness and ends with acts of kindness.”

The obligation to care for the vulnerable, including the poor, orphans, widows, and stranger, is repeatedly stressed, even when this requires exceeding the formal law:

- **Tractate Sukkah 49b**: “Acts of kindness are greater than charity, for charity is done with one’s money, while acts of kindness are done with one’s person, money, and soul.”
- **Tractate Baba Metzia 31a:** “If you see the donkey of one who hates you lying under its burden, you shall refrain from leaving him with it; you shall surely help him (Exodus 23:5). From this, the Sages derive the obligation to assist others in relieving their burdens, even if it requires effort or inconvenience. This principle extends beyond animals to any situation where one can help alleviate another’s suffering or difficulty.”

It may be claimed that the injunctions contained in these passages cease to apply in times of violent conflict with an enemy. However, arguably, they are presented without qualification and therefore continue to apply even under the most adverse circumstances. Perhaps most tellingly, the Talmud elaborates at length on the biblical commandment to “love your neighbour as yourself” (Leviticus 19:18), quoted above, stressing its foundational force. As Rabbi Hillel said (Schwarz 2012):

- **Tractate Shabbat 31a**: “What is hateful to you, do not do to your neighbour. The rest is commentary; go and learn.”

## Talmudic Responses to Conflict: The Case of the Pursuer and the Intruder

In addition to these general principles, there are multiple passages in the Talmud that refer directly to circumstances where violent conflict either threatens or actually occurs. Two metaphors are invoked to analyse challenges that arise in these settings: those of the “pursuer” and the “intruder.” The discussions appear in the Sanhedrin tractates dealing with civil and criminal law, rather than in relation to more general principles of warfare and, accordingly, their relevance to the Israeli actions in Gaza may be debated. Furthermore, their application in the current conflict is obviously ambiguous, since at different times both the Israelis and Hamas have occupied the roles of pursuer and pursued, and intruder, making a single clear ethical inference regarding the actions of the IDF impossible. Nonetheless, their historical prominence and influence and their repeated application in the present setting means that they must be considered in detail.

(i) The pursuer (“rodef”)

In Sanhedrin 73a The Talmud introduces the principle of the pursuer: if someone is actively pursuing another person to kill or harm them, it is a duty to stop the pursuer—even if this requires lethal force.

The text arises as a commentary on a passage in Deuteronomy 25:12, which, referring to a domestic dispute, states: “You shall cut off her hand; your eye shall not pity.” The Talmud interprets this verse as permitting intervention to save a victim from harm, even if it means using force against the pursuer. Specifically, it states:

- **Sanhedrin 73a**: “The Sages taught yet another baraita on the topic of a pursuer: One who pursues another to kill him; or pursues a male to sodomize him; or pursues a betrothed young woman to rape her; or pursues a woman who is forbidden to him by a prohibition … all these people are to be saved at the cost of the life of the transgressor. But with regard to a widow who is being pursued by a High Priest, or a divorcé … they are not saved at the cost of the rapist’s life, because these unions are subject only to a mere prohibition.”

The complexity of the circumstances is emphasized by the assertion that if a “sin” had already been committed with the betrothed young woman she should not be saved at the cost of the rapist’s life. In addition, if there is one to save her, that is, if there is another way to save the betrothed young woman that does not involve killing the rapist, she is not saved at the cost of his life. Rabbi Yehuda says:

- **Sanhedrin 73a:** “Also, if the betrothed young woman says to those who come to rescue her: Let the rapist be, she is saying this so that he should not kill her, and therefore the rapist is not killed.”

This complexity, and the need to analyse the details of specific contexts, is explicitly recognized by the Rabbis:

- **Sanhedrin 73a:12**: “The Gemara asks: Why do I need all these different cases? Why does it not suffice to offer one example from which all the others can be derived? The Gemara answers: These cases are all necessary … [otherwise] one might say that the victim … is saved at the cost of his attacker’s life, because it is not his way to engage in intercourse with a man, and so he would suffer excessive embarrassment and pain were he not saved. But a young woman, whose natural way is to engage in intercourse with a man, one might say that her attacker may not be killed.”

The Talmud elaborates on the ethical limits of this principle: **t**he pursuer must have a clear intent to kill or cause severe harm. If the pursuer’s intent is uncertain, the use of lethal force is not permitted; **t**he danger must be immediate. If the threat is not imminent, other means of preventing harm must be sought; the response must be proportional to the threat. One may only use the minimum force necessary to stop the pursuer; and care must be taken to avoid harming innocent bystanders while intervening to stop the pursuer.

In relation to the questions posed by contemporary circumstances, the Talmudic debate emphasizes the need for extreme caution. The principle of the *Rodef* is not a blanket licence to kill but is subject to strict ethical and legal constraints. The goal is to preserve life, not to take it unnecessarily. The Talmudic discussion in Sanhedrin 73a reflects a nuanced approach to self-defence and the protection of others, balancing the imperative to save lives with the need to minimize harm and act justly.

Further, as stated, an unequivocal application to the conflict in Gaza is not possible because of the multiple roles played by all the protagonists. In particular, the IDF cannot adduce the concept of the rodef to justify its attacks on the people of Gaza without itself becoming subject to its own limitations.

(ii) The intruder

In an accompanying discussion, Sanhedrin 72a considers a burglar breaking into a home, presuming that the intruder may use deadly force if confronted. The home-owner is allowed to kill the intruder pre-emptively to protect their life and property. However, here too there are strict limits. If it becomes evident that the intruder poses no direct threat to life— e.g. if they are fleeing or unarmed—the home-owner may not kill them.

The principle espoused here reflects an ethical tension between protecting property and the foundational value of the sanctity of human life, as well as the need to consider the contextual details, including even the time at which the offence occurred:

- **Sanhedrin 72a:16:** “If the sun is risen upon him, there shall be blood shed on his account. A question may be raised: But did the sun rise only upon him? Rather, these words must be understood as follows: If the matter is as clear to you as the sun that the burglar is coming to you in peace, do not kill him. But if you are not sure about his intentions, arise and kill him.”

Here too, the applicability of this analysis to our question is limited by the simple fact that the inhabitants of the territory of Israel and the IDF are simultaneously both perpetrators and victims of an “intrusion.” Nonetheless, it can be seen that the Talmud through this discussion emphasizes that even in cases that may at first sight seem straightforward one must not take a life unnecessarily. The use of lethal force is only permitted when there is a reasonable, imminent fear for one’s own life or the lives of others. If it is possible to prevent a crime or offence being committed without the taking of a life this will always be the preferred course of action.

(iii) Indirect harm and collateral damage

An additional discussion is more unambiguously relevant to the IDF actions in Gaza. The Talmud examines the concept of indirect harm. While one may be liable for initiating the circumstances that gave rise to an injury, according to the sages they are only held accountable for the damage if they failed to take reasonable precautions.

This principle is of relevance to contemporary discussions about collateral damage. The Talmud is clear that if harm to civilians is foreseeable but not intentional, ethical liability depends on whether all reasonable precautions were taken to prevent it.

An example considered relates to the damage caused by a spreading fire. If the harm is foreseeable, one must act to mitigate it, even at significant cost. Harm to bystanders can only be ethically permissible if it is unintended and unavoidable while addressing a pressing threat. As usual, the Rabbis’ reflections focus on fine conceptual distinctions:

- **Bava Kamma 60a:** “If a fire breaks out and spreads due to the wind, is the one who lit the fire liable for the damage?”

However, their conclusions are clear-cut:

- **Bava Kamma 60a:8**: “If someone brings on a fire which consumes wood, stones, or earth, he would be liable, as it is written (Exodus 22:5): ‘If fire breaks out and catches in thorns so that the stack of corn, or the standing corn, or the field is consumed, he who starts the fire must make restitution.’”

As with the other discussions, while at first glance this one may appear to be distant from the great questions of conduct in lethal combat, the larger context, which includes reference not just to domestic fires but also to epidemics and famines, makes clear that the conclusions relate directly to the devastating effects of war (Levinas 2019). For this reason the affirmation that ethical responsibilities are not limited or abrogated by disruptive events such as ones described carries immediate and strong relevance for the events in Gaza. According to the Talmud, a military response must be proportional to the threat posed by the enemy. Those involved in such a response are obliged to avoid harm to non-combatants, even when targeting a *rodef*.

## Responses to Common Ethical Arguments Concerning the Actions of the IDF

Taking into account these various and sometimes conflicting considerations, what then, is the Talmudic view about the justifications that are commonly provided for the tactics used by the IDF in its assault on Gaza?

As discussed, the biblical texts can be read as offering a variety of putative justifications for aggressive military tactics, including the existence of a divine covenant, commandments to drive out inhabitants, the right of self-defence, an obligation to eradicate evil, protection of the Promised Land, retribution, and the assumption of divine protection for the People of Israel. Regardless of beliefs that may be aligned to such biblical passages, the Talmudic and rabbinic literature contains a great deal of additional material relevant to many of the basic issues which have arisen in the current conflict and have been the subject of debates about the morality of the policies and actions of the IDF. In addition, the discursive structure of the Talmud in many ways reverses that of the Bible, throwing into question some of its key precepts. Where the Bible is structured around divine speech, moral exhortations, and apodictic imperatives the Talmud presupposes a human conversation as the pathway to truth and ethical propriety. This is the case already in the Mishnah, of which a condition of possibility is rabbinic interpretation generating normative, but not univocal, legal categories. Here, ethical decisions are no longer based on putative divine fiats but rather reflect communal reasoning aimed at supporting human responsibility. In the Gemara this is even more clearly the case, with the explicit transition to a conversation that is heteroglossic, recursive, and dialogical, with the repeated evocation of the complexity of interactive experiences and the deliberate preservation of questions, contradictions, and aporia (Boyarin 1990, 1993, 1994; see also Levinas 1979 and Levinas and Hand 1997).

As also mentioned, the attempts at justification of Israeli actions, including attacks on children and other civilians, destruction of health and educational facilities, and the denial of food and other aid, do not just depend on the authority of the Bible but also seek to call on more conventional contemporary ethical reasoning. Some of the key questions addressed in these discourses have already been mentioned but others require specific attention, including the need to protect civilian populations and the question of proportionality, responses to the use of human shields, and the right to self-defence. These have been under discussion by the Israeli government and in the international political and diplomatic communities as well as in vigorous civil society debates around the world. The Talmudic corpus provides important guidance in relation to all of these questions.

(i) Civilian protection as a moral imperative and proportionality

The biblical texts often prioritize military objectives and collective survival, which might permit greater civilian harm when justified by divine command or existential threats. By contrast, the Talmudic interpretations significantly temper this stance by introducing procedural and ethical safeguards. Further, while the Bible’s exhortations—such as those to destroy Amalek or Canaanite cities—often reflect a high level of violent intent, the Talmud’s procedural restrictions and proportionality principles impose significant ethical limitations.

Indeed, following this strategy, the Rabbis spiritualize the command to destroy Amalek as a fight against evil or immorality, rather than interpreting it a literal injunction to engage in total war. For example, Sanhedrin 20b emphasizes the need for legal and procedural oversight before engaging in war, requiring a prophetic mandate and similarly, regarding the Canaanites, the Talmud and later commentators frame the commands as historically bounded and contingent. In addition, Sanhedrin 56a introduces the Noahide Laws, which establish universal ethical standards applying to all humanity and tempering the Bible’s seemingly absolute commands. In a similar manner, as previously discussed, the authors of Bava Kamma 60a and later commentators stress minimizing harm and avoiding unnecessary suffering, even in justified conflicts.

This difference in approach between the biblical injunctions and the Talmudic guidance is fundamental and applies generally. The text in the Bible is often brief and unnuanced and is potentially open to interpretations that ignore the complexities of local conditions, as well as avoiding a detailed process of dialogue prior to practical decision-making. The Talmud, on the contrary, is heavily focused on elaborating the multiple possible meanings of a text, which stress the need for careful analysis and open conversation in order to achieve a consensus position.

The Talmud certainly permits lethal force in cases of imminent danger, while continuing to emphasize the importance of proportionality. However, if a threat can be neutralized without killing the aggressor—such as through wounding or disarming—the injunction becomes more definitive and one is obligated to pursue the less extreme option (Bava Kamma 28a). Unnecessary killing, even in self-defence, can render the defender liable.

Moreover, the Talmud’s overwhelming emphasis on *pikuach nefesh* mandates extraordinary efforts to protect civilian populations, even in the midst of conflict. The concern for limiting collateral damage (Bava Kamma 60a) demands that responses to threats always remain proportionate and avoid unnecessary destruction. Applying this principle generates strong injunctions to avoid tactics that indiscriminately harm infrastructure essential to civilian survival, such as hospitals, water supplies, and food distribution centres, at almost any cost.

(ii) Human shields

The alleged use by Hamas of human shields has been repeatedly cited by Israel as a justification for attacks on civilian populations. It is claimed that if a non-combatant, who may be a child, is in the proximity of a Hamas fighter there is no moral obligation on the IDF to protect that person because the responsibility for their welfare and the danger in which they have been placed lies with the enemy. This argument has been used to justify attacks on apartment blocks, refugee encampments, hospitals, and even United Nations enclosures, where in some cases literally hundreds of civilians have been killed to achieve the death of a single enemy operative.

As with the rodef and intruder arguments this one is difficult to apply because there is evidence that Israel itself uses human shields, sometimes deliberately employing civilians as an advance party in dangerous conditions (Associated Press 2025; Anonymous 2025). However, because this moral question is so prevalent it is important to consider it here.

In relation to the question of human shields there is also a likely divergence between the perspective that might be inferred from the biblical texts and the more nuanced analyses of the Talmud. In fact, the Bible does not explicitly address the concept of human shields and so is largely silent on the matter, although it demonstrates little regard for civilian “collateral” damage. In contrast, Talmudic ethics emphasizes at length individual accountability and stresses a moral imperative to protect innocent people, even when they are used as shields. It thereby requires that strict steps be taken to limit harm to such people and assigns moral culpability to those who fail to do so.

The Talmud emphasizes that one may not take an innocent life, even to save one’s own life:

- **Sanhedrin 74a:** “If a man says to you, ‘Go and kill so-and-so, and if not, I will kill you,’ let him be killed rather than transgress.”
- **Pesachim 25b:** “One may not save their own life by shedding another’s blood.”

An individual cannot determine that his or her own life is more valuable than another’s. This principle underscores the ethical prohibition of intentionally harming non-combatants, regardless of the tactical situation.

Not surprisingly, the Talmud unequivocally condemns the use of human shields, which would be seen as a form of coercion. Multiple texts indicate that those who adopt such tactics incur a heavy moral responsibility. Notwithstanding this, those responding to the threat are obliged to take extraordinary measures to protect the lives of the people thereby endangered. This is stated explicitly by the sixteenth century sage, Rabbi David ben Zimra (Radbaz):

- **Responsum 3:627:** “If the only way to kill the rodef (pursuer) is by also killing an innocent person, and the innocent person is certain to die, it is forbidden to do so” (Jachter 2023; Morell 2003).

The civilian victims unequivocally remain under the protection of the principle of *pikuach nefesh*.

(iii) Prohibition of collective punishment

Prohibition of collective punishment is a strong injunction shared by the Bible and the Talmud. In his dialogue with God over Sodom, Abraham pleads:

- **Genesis 18:23–32:** “… Abraham drew near and said, ‘Will you indeed sweep away the righteous with the wicked? ... Far be it from you to do such a thing, to put the righteous to death with the wicked, so that the righteous fare as the wicked! Far be that from you! Shall not the Judge of all the earth do what is just?’”

Both the Bible and the Talmud agree emphatically on the response:

- **Sanhedrin 27b:** “It is as the Sages taught in a baraita: ‘The fathers shall not be put to death for the children, neither shall the children be put to death for the fathers; every man shall be put to death for his own sin’ (Deuteronomy 24:16). Why must the verse state this first clause? If it is to teach that the fathers shall not be put to death for the sin of the children, nor shall the children be put to death for the sin of the fathers, this is unnecessary, as it is in any event stated: ‘Every man shall be put to death for his own sin’.”
- **Sanhedrin 11a**: “A judge must not punish one person for another’s sin.”

These passages affirm an unequivocal prohibition of collective punishment. They emphasize that only individuals who commit sins or crimes can be held accountable, and that innocent people (including family or other community members) cannot be punished for the actions of others. This is a strong obligation that is repeated multiple times in both the Bible and the Talmud.

(iv) Application to the right to self-defence

The biblical tradition regards a right to self-defence as fundamental and conceptualizes this in black and white terms. By contrast, the Talmudic and rabbinic traditions, while recognizing and supporting a fundamental right to defend oneself, elaborate multiple qualifications and limits to ensure this right is not used as a device to employ excessive violence.

In Sanhedrin 72a it is clearly stated that “If someone comes to kill you, rise and kill him first.” Indeed, there are occasions where self-defence actually becomes a moral duty. However, multiple passages, in this tractate and elsewhere, explicate the precise conditions which need to be satisfied for the actions to be justified as self-defence.

In imposing these conditions, the Talmud is intentionally invalidating the concept of private vengeance through the “avenger of blood” system, replacing it with judicial oversight (Sanhedrin 35a) and moving toward an emphasis on proportionality and procedural justice. In place of biblical passages that appear to draw on self-defence as a straightforward justification for violence that is really based on vengeance, the Talmud imposes stringent procedural safeguards, always subject to the emphasis on the sanctity of life. This is a critical evolutionary development in Jewish ethics.

In the Talmudic system, for a violent act to be considered self-defence, there must be an immediate and direct threat. For instance, in cases of an intruder breaking into one’s home (Sanhedrin 72a-b), lethal force is permitted only if it is clear that the intruder poses a deadly threat; if there is doubt or an alternative means to neutralize the danger, lethal action in self-defence is not justified. In addition, the harm against which one is seeking to protect oneself must be intentional and deliberate. Harm without malice—such as in cases of negligence—does not justify a lethal response. This anti-utilitarian distinction between intentional and accidental acts (Makkot 7a) is fundamental.

Self-defence may include the defence of others, as required by the collective responsibility to prevent harm to the vulnerable (Sanhedrin 73a). Therefore, on occasion, there may even be an obligation to intervene to protect innocent lives, even if doing so requires lethal force. Where acts of self-defence are undertaken, the overriding framework of *shalom* (peace) and the sanctity of life can never be undermined (Pirké Avot 1:12**)**.

However, even when self-defence is justified, the responsibility to minimize harm remains paramount. Aggression disguised as self-defence is a serious ethical and legal violation. For instance, someone who provokes a confrontation and then claims self-defence may be held liable. Misuse or abuse of self-defence is culpable behaviour. If someone kills another under a false or mistaken claim that it was necessary for self-defence, he or she incurs liability, potentially subject to serious punishment or exile:

- **Makkot 7a**: “One who kills a person unintentionally goes into exile.”
- **Sanhedrin 9b: “**One who testifies against another to have them killed ... is a shedder of blood.”

Maimonides states this even more clearly:

- **Mishneh Torah, Hilchot Rotzeach 1:13:** “One who kills a person who was not a pursuer is a murderer and must be executed ... even if they acted in error.”

In summary, the Talmudic texts reject simplistic views of self-defence, affirming that this is not an unqualified right but a responsibility bound by strict ethical limits, that alternatives to lethal force should always be prioritized, and a careful evaluation of intent, necessity, and proportionality cannot be circumvented.

## Injunctions in the Talmudic and Extended Rabbinic Literature About the Need to Ensure Protection of Children

The protection of children and the prevention of harm is one of the most fundamental and enduring precepts of the Jewish tradition. The entire corpus of Jewish knowledge and ethics is replete with injunctions to protect children and shield them from harm in all imaginable circumstances.

The Bible itself emphasizes the fundamental nature of this obligation. Children are a divine gift and responsibility and must be protected from violence:

- **Psalm 127:3:** “Behold, children are a heritage from the Lord, the fruit of the womb a reward.”
- **Isaiah 49:15:** “Can a mother forget the baby at her breast and have no compassion on the child she has borne? Though she may forget, I will not forget you!”
- **Isaiah 1:17:** “Learn to do right; seek justice. Defend the oppressed. Take up the cause of the fatherless; plead the case of the widow.”

The subsequent literature elaborates these themes in detail. For example:

- **Shabbat 119b**: “The world only exists because of the breath, reciting Torah, of schoolchildren … The breath of adults, which is tainted by sin, is not similar to the breath of children, which is not tainted by sin … Any city in which there are no schoolchildren studying Torah, they destroy it. Ravina said: They leave it desolate.”

As always, the principle of *pikuach nefesh* overrides nearly all commandments, which further supports the moral obligation to protect children, including in times of war (Mishnah Yoma 8:6, Yoma 85b). This principle extends to war and conflict situations, where the preservation of innocent life—including that of children—is paramount. The post-Talmudic literature is also replete with discussions about the importance of children (Heschel 1976a, 1976b; Sacks 2007).

## Conclusions 1: The Bible or the Talmud?

The relationship between Talmudic principles and those embedded in pre-Talmudic thought, specifically the Bible, entails both continuity and divergence. The Bible provides the foundational texts for Jewish law and ethics but its interpretation within the Talmudic corpus often transforms its applicability, emphasizing more refined and sophisticated ethical and legal frameworks. The comparison exposes important distinctions, particularly regarding war, self-defence, conflict resolution, and the treatment of non-combatants.

The Bible prioritizes collective survival, justice, and divine command, often allowing harsh measures in war. It also provides the foundational principles of justice, self-defence, and the sanctity of life, often reflecting the immediacy and pragmatism of its historical context. As previously mentioned, it also contains many calls for compassion, justice, and care for the oppressed (e.g., Isaiah 1:17, Micah 6:8), which are frequently ignored by those tendentiously seeking passages that appear to offer justifications for acts of violence.

Many of the later commentaries, in the Talmud and other works, contextualize the pronouncements of the Bible and extend and deepen those that at first sight may seem peremptory and of limited flexibility. Local references are often transformed metaphorically or metonymically from categorical injunctions into generalizable principles: for example, as mentioned, “Amalek” becomes a reference to the principle of evil rather than a call to commit mass murder.

In a similar manner, the Bible often prioritizes “justice” over reconciliation. For example, Deuteronomy 7:2 commands the Israelites to destroy the Canaanites and “show them no mercy,” reflecting a view that peace is subordinate to divine justice; and Deuteronomy 20:10–14 permits the killing of men in a conquered city and allows women, children, and livestock to be taken as plunder. By contrast, the Talmud (Sanhedrin 56a**)** introduces ethical standards that apply to all people and nations, emphasizing the protection of innocents and limits on violent behaviour and the use of lethal force. Later rabbinic literature extends further the principle of avoiding unnecessary harm: for example, Shabbat 151b states, “Whoever has mercy on others, Heaven will have mercy on them,” universalizing the ethic of compassion.

The differences reflect a fundamental divergence between the logical strategies of two bodies of text. It can now be seen how important this is for the contemporary ethical debates. In the Bible, the discourse involves a singular, transcendent voice issuing commands and theological declarations. It presents itself as a coherent, closed text with a claim to ultimate truth and authority. While it undoubtedly contains ambiguity and multiple genres, its structure tends toward narrative unity and theological closure. Biblical law is given in apodictic form and the logic is directive and declarative. There is little or no reflexivity, and—despite the existence of passages that are manifestly contradictory—there is no acknowledgment of contending points of view. By contrast, Talmudic logic employs dialectical, layered, case-based discussions that invariably entail multiple perspectives in a non-linear, recursive manner. Discussions are often inductive or rhetorical, employing multiple tropes, and are therefore inherently open to reinterpretation and continuing debate. Contradiction and ambiguity are not regarded as problems to be eliminated but rather as sources of creative difference that generate novel ideas. Ethical norms are not given all at once but worked out in relationships, through listening, challenging, and refining. Ethics is communal, contested, and evolving—a process rather than a product. Similarly, the ethical subject in the Talmud is not just a rule-follower but an interpreter, responsible for making meaning in specific contexts. Interpretation is a moral act—how we read, what voices we privilege, and how we resolve tensions have ethical consequences. Jewish identity is thereby characterized as inherently dialogical and interpretive—not dogmatic or purely legal.

In sum, in place of the declarative assertions of the Bible, the Talmud substitutes rational procedures and universalizable arguments to underpin ethical decision-making. In place of imperatives, the Talmudic texts are grounded in deliberation, dialogue, and detailed analysis of practical circumstances. From this point of view, therefore, on the complex subjects of the ethics of conflict, war, and violence, the deliberations in the Talmud and rabbinic texts carry overwhelming moral weight.

## Conclusions 2: The Talmudic Responses to Ethical Issues in Conflicts

Judaism cannot be encapsulated in a single set of principles, beliefs, or claims that constitute its “essence” or central core; indeed, it cannot be characterized as a single body of unambiguous rules and beliefs. Instead, as this analysis shows, it is more accurate to conceptualize it as a seething, evolving culture of language and reason in a context of values that allows for multiple points of view and stimulates and encourages dissent as much as consensus. It is a complex body of thought that incorporates and stimulates dynamic dialogues about ethics in a manner that is self-reflective and directed towards an infinite field of applications and judgments.

Nonetheless, in spite of this commitment to diversity, universal themes do exist that are central to all Jewish thought. This essay has described some of these as they relate to the ethical conduct of war. These in turn allow important inferences to be drawn about how key issues arising out of the conflict in Gaza appear from the Talmudic and rabbinic perspectives.

Our analyses of the Talmudic and later texts have identified eight substantive propositions regarding the conduct of war:

- First, civilian protection and the sanctity of life: The principle of *pikuach nefesh* is central to Talmudic ethics. This mandates that every effort must be made to protect civilians, even in the midst of war.
- Second, while Talmudic texts regard the use of human shields as a grave moral violation, as it places innocent lives at risk, a heavy moral responsibility for using human shields falls on those who adopt such tactics.
- Third, the Talmud requires proportionality and precision. Airstrikes or other actions that risk significant civilian casualties would only be permissible if there is an immediate, intentional, and critical threat to life. Harm caused to innocents must be minimized, and the response to aggression should not exceed what is necessary to neutralize the threat. Actions that cause significant collateral damage can only be justified if no other options exist to prevent greater harm.
- Fourth, the Talmud prioritizes peace and reconciliation, even amidst conflict. Efforts to de-escalate violence and protect civilians are paramount.
- Fifth, the Talmud emphasizes addressing root causes of conflict. Efforts to achieve a sustainable peace, such as addressing political grievances and improving living conditions for Gazans, are consistent with its teachings.
- Sixth, the Talmud places moral responsibility on aggressors who create such conditions but also holds defenders accountable for their actions.
- Seventh, the principle of collective responsibility suggests a duty to alleviate suffering, even among perceived adversaries, whenever possible.
- And finally, Talmudic ethics demand accountability for harm caused, whether intentional or inadvertent.

As translated from broad principles and fundamental commitments of Jewish ethics to the specifics of the war in Gaza, the implications are clear and inescapable:

- The targeting of civilians and in particular, the large-scale murder of children, is directly contrary to the most elementary precepts of Jewish ethics.
- Collective punishment, exemplified in the onslaught on Gaza, is unambiguously prohibited by Jewish law.
- The obstruction of deliveries of food, humanitarian aid, medical care, and safe evacuation of civilians blatantly contravenes the obligation to prioritize life.
- The refusal to resolve political grievances through reconciliation and negotiation contradicts the Talmud’s vision of peace.
- The arguments that attacks on civilians and the widespread devastation of homes, hospitals, schools, public spaces, agricultural land are justified by the right to self-defence are inconsistent with Jewish law and ethics.
- The claim that such actions are justified by the alleged or real use of human shields directly contradicts Talmudic teachings.
- The failure to take special precautions to protect children stands in opposition to fundamental assumptions underlying all Jewish ethics.

## Conclusions 3: Consistency of the Actions of the IDF in Gaza with Judaic Ethics

In this essay, we set out to address whether the specific actions of the Israeli government and the IDF are consistent with the actual beliefs, traditions, and teachings of Judaism. The conclusion is straightforward and compelling: Israeli government policy and the conduct of the IDF diverge in fundamental ways from the fundamental and constitutive ethical commitments of Judaism.

Understood in the context of the fullness and complexity of Jewish thought, the policies and actions pursued by the current Israeli regime bear little relationship to the ethical and philosophical culture that has been established and maintained over the millennia. Put more bluntly, the current regime cannot be seen as a valid representative of the Judaic tradition or of Jewish thought. Neither its actions nor the arguments it advances to justify them is supported by the authoritative texts or can be regarded as consistent with the deep and enduring body of knowledge and wisdom that is constitutive of Jewish culture.

This conclusion raises many questions of its own, some of which are wide ranging. They include: the degree to which criticism of the Israeli government and its policies can be appropriately attributed to antisemitism (including debates over competing definitions of this term); the general character of the Israeli state, which proclaims itself to be the “Jewish State,” along with the Israeli government and politics; the role and place of Israel in relation to international law and the ethical frameworks applied to other nation-states in the world community; and the self-identity of the Jewish people and how they understand their relationships and obligations to other cultures, their own history, and to each other.

These questions undoubtedly pose some significant challenges. But these are, of course, not the first such challenges in the history of the Jewish people. Indeed, today it is hard not to hear an echo of the cry of the prophet Jeremiah, in similarly tragic times:

- **Jeremiah 8:21–22** “For the brokenness of the daughter of my people I am broken; I mourn, and horror has taken hold of me. Is there no balm in Gilead? Is there no physician there? Why then has the healing of the daughter of my people not come?”

## Data Availability

Not applicable.

## Footnotes

1 Technically, the “descendants of Abraham” in both the Hebrew Bible and the Koran include both Isaac, the ancestor of the people now known as Jewish Israelis, and his brother, Ishmael, the ancestor of the Arab people whom we now call Palestinians. This obvious “biblical fact” is not usually acknowledged by those who argue for the mandate.

2 In what follows, quotations from the Babylonian Talmud will mostly (but not entirely) be drawn from the Sefaria version of the text, originally translated by Rabbi Adin Steinsaltz into modern Hebrew and then subsequently into English in the form now known as the *William Davidson Talmud* (2017). Other quotations are provided from Rodkinson 1918.
